# Current in the Protein Nanowires: Quantum Calculations of the Base States

**DOI:** 10.1186/s11671-016-1269-0

**Published:** 2016-02-09

**Authors:** Anatol D. Suprun, Liudmyla V. Shmeleva

**Affiliations:** Department of Theoretical Physics, Faculty of Physics, Taras Shevchenko National University of Kyiv, Volodymyrska Street, 64/13, Kyiv, 01601 Ukraine

**Keywords:** Protein molecule, Numbers of filling, Injected electron, External field, Current density

## Abstract

It is known that synthesis of adenosine triphosphoric acid in mitochondrions may be only completed on the condition of transport of the electron pairs, which were created due to oxidation processes, to mitochondrions. As of today, many efforts were already taken in order to understand those processes that occur in the course of donor-acceptor electron transport between cellular organelles (that is, between various proteins and protein structures). However, the problem concerning the mechanisms of electron transport over these organelles still remains understudied. This paper is dedicated to the investigation of these same issues.

It has been shown that regardless of the amino acid inhomogeneity of the primary structure, it is possible to apply a representation of the second quantization in respect of the protein molecule (hereinafter “numbers of filling representation”). Based on this representation, it has been established that the primary structure of the protein molecule is actually a semiconductor nanowire. In addition, at the same time, its conduction band, into which an electron is injected as the result of donor-acceptor processes, consists of five sub-bands. Three of these sub-bands have normal dispersion laws, while the rest two sub-bands have abnormal dispersion laws (reverse laws). Test calculation of the current density was made under the conditions of the complete absence of the factors, which may be interpreted as external fields. It has been shown that under such conditions, current density is exactly equal to zero. This is the evidence of correctness of the predictive model of the conductivity band of the primary structure of the protein molecule (protein nanowire). At the same time, it makes it possible to apply the obtained results in respect of the actual situation, where factors, which may be interpreted as external fields, exist.

## Background

For some time past, the investigations of physical properties of proteins (particularly, such as transfer of energy or transfer of charge [1]) are performed taking into account their actual structure [2–10] with increasing frequency. In effect, amino acid inhomogeneity [2, 3], discreteness [4, 5], finite length of the α-spiral section [6], and other factors [7–10] are inherent in general to the structures containing carbon [11–13]. Particularly, average-electron structure and average-nuclear structure of the protein molecule were analysed in detail in article [14]. The goal of the analysis within the present article was to investigate the type of crystallinity of the primary structure of these molecules, as well as to investigate the possibility of performance of the electron transport through this structure to a mitochondrion. Hypothesis suppositions concerning the semiconductor type of crystallinity of the primary structure of the protein molecule, which were expressed long ago [15–20], were approved. In fact, it is a semiconductor nanowire having the average-oxygen structure of the electron configuration, as well as the average-nitrogen composition of the nuclear subsystem (along with the hydrogen compensators of the charge deficit). The average-oxygen electron structure makes it possible to implement the model, in accordance with which such protein nanowire has five energy bands, one of which is a conductivity band, while other energy bands are valence bands. The average-nitrogen nuclear subsystem makes it possible to select a relevant basis for transformation into the numbers of filling representation in the form of wave functions of the single-electronic nitrogen ion (having the charge number *z* = 7). In addition, reason of the electron transport over the protein nanowire was clear, that is, the reason of actual existence of a microcurrent through the primary structure of protein in the absence of external fields. This reason is connected with the presence of residual electrostatic long-range field, which is connected with the inhomogeneous pattern of the system (this pattern is associated with the presence of radicals in the amino acids). It has been established that availability of not identical radicals has no any substantial influence upon the electron configuration of the protein molecule, because these radicals do not take part in the creation of the primary structure. However, at the same time, availability of such radicals adds substantial complexity to the elementary cell, and this fact has influence upon the structure of the spectrum of electronic states, particularly, upon the energy structure of the conductivity band. The structure of this band is the main subject of scientific research within the present article. And this subject of research is based on the fact that protein molecule in the zeroth-order approximation is considered in the nitrogen-oxygen model [14] in the conditions of the absence of all external influences, including those that are connected with the amino acid inhomogeneity of the protein molecule. It has been shown that in such conditions, which are idealised in respect of the external field, the electron, which was injected into the conductivity band, does not create any current. This is the evidence of correctness of the energy structure of the conductivity band, which was determined in this article.

## Methods

### Description of an Electron Injection to the Conductivity Band as an Excited State of a Protein Molecule

Energy operator of the primary structure of protein molecule (protein nanowire) in accordance with the numbers of filling representation was formulated in article [14] as follows:

1 $$ \begin{array}{c}\widehat{H}={\displaystyle \sum_{f\mathbf{n}}{\varepsilon}_f{b}_{f\mathbf{n}}^{+}{b}_{f\mathbf{n}}}-{\displaystyle \sum_{f\mathbf{n}}{\displaystyle \sum_{\mathbf{l}}{\displaystyle \sum_{g\kern0.1em \mathbf{m}}{}^{//}Q_{\mathbf{n}\kern0.1em \mathbf{l}\kern0.1em \mathbf{m}}^{\kern0.1em fg}\kern0.1em {b}_{f\mathbf{n}}^{+}{b}_{g\kern0.1em \mathbf{m}}}}}+\\ {}+\frac{1}{2}{\displaystyle \sum_{f\mathbf{n}}{\displaystyle \sum_{g\kern0.1em \mathbf{m}}{\displaystyle \sum_{f^{\prime}\kern0.1em {\mathbf{n}}^{\mathbf{\prime}}}{\displaystyle \sum_{g^{\prime}\kern0.1em {\mathbf{m}}^{\mathbf{\prime}}}{V}_{\mathbf{n}\mathbf{m}{\mathbf{n}}^{\mathbf{\prime}}{\mathbf{m}}^{\mathbf{\prime}}}^{fg\kern0.1em {f}^{\prime}\kern0.1em {g}^{\prime }}\kern0.1em {b}_{f\mathbf{n}}^{+}{b}_{g\kern0.1em \mathbf{m}}^{+}{b}_{f^{\prime}\kern0.1em {\mathbf{n}}^{\mathbf{\prime}}}{b}_{g^{\prime}\kern0.1em {\mathbf{m}}^{\mathbf{\prime}}}}}}}+{\displaystyle \sum_{f\mathbf{n}}{\displaystyle \sum_{g\kern0.1em \mathbf{m}}{\tilde{W}}_{\mathbf{n}\mathbf{m}}^{f\kern0.1em g}\;{b}_{f\mathbf{n}}^{+}{b}_{g\kern0.1em \mathbf{m}}}}\kern0.5em .\end{array} $$

Double slash primes over the summation symbols point to the absence of those summands, where **n** = **l** = **m**. Matrix elements, which are included to this operator, have the following definitions *ε*_*f*_≡ − (*z*/*n*_*f*_)^2^*ε*_*R*_ . Subscript *f* of the energy *ε*_*f*_ points to the standard set of quantum numbers: *f* = {*n*_*f*_, *l*_*f*_, *m*_*f*_}, where *n*_*f*_ = 1, 2, 3, … is the main quantum number, which belongs to the set *f*, *l*_*f*_ = 0, 1, 2, … , *n*_*f*_ − 1 is the orbital quantum number, while *m*_*f*_ = 0, ± 1, ± 2, … , ± *l*_*f*_ is the azimuthal quantum number of the same set. Similar definitions exist for the sets *g*, *f*′, and *g*′ as well. ${\varepsilon}_R\equiv \frac{m_e{e}^4}{2\;{\hslash}^2}$ is the Rydberg energy, *z* is the charge number (for the average-nitrogen basis *z* = 7). This energy is the same as the energy of the single-electronic nitrogen ion, while the total amount, which holds this energy, has physical significance of the considered system, within which any interactions between electrons and nuclei are absent. ${Q}_{\mathbf{n}\kern0.1em \mathbf{l}\kern0.1em \mathbf{m}}^{\kern0.1em fg}\equiv \left\langle {\varphi}_{f\mathbf{n}}\left(\mathbf{r}\right)\right|z{e}^2/\left|\mathbf{r}-\mathbf{l}\right|\left|{\varphi}_{g\kern0.1em \mathbf{m}}\left(\mathbf{r}\right)\right\rangle$ is the modulus of the matrix element of the energy of interaction between an electron and all nuclei, which are situated in the spatial positions: **l**. Averaging is made in accordance with the quantum understanding over the hydrogen-like wave functions of the single-electronic nitrogen ion: *φ*_*f***n**_(**r**), *φ*_*g* **m**_(**r**). They are centred in respect of the spatial positions **n**, **m**; that is, they have the following property: *φ*_*f***n**_(**r**)≡*φ*_*f*_(**r** − **n**). This property makes it possible to formulate simplified representations for the matrix elements, particularly, such representations, which only depend on the difference **n** − **m**. ${V}_{\mathbf{n}\mathbf{m}{\mathbf{n}}^{\mathbf{\prime}}{\mathbf{m}}^{\mathbf{\prime}}}^{fg\kern0.1em {f}^{\prime}\kern0.1em {g}^{\prime }}\equiv \left\langle {\varphi}_{f\mathbf{n}}\left({\mathbf{r}}_1\right){\varphi}_{g\kern0.1em \mathbf{m}}\left({\mathbf{r}}_2\right)\right|{e}^2/\left|{\mathbf{r}}_1-{\mathbf{r}}_2\right|\left|{\varphi}_{f^{\prime}\kern0.1em {\mathbf{n}}^{\mathbf{\prime}}}\left({\mathbf{r}}_2\right){\varphi}_{g^{\prime}\kern0.1em {\mathbf{m}}^{\mathbf{\prime}}}\left({\mathbf{r}}_1\right)\right\rangle$ is the modulus of the matrix element of the electronic interaction energy, while ${\tilde{W}}_{\mathbf{nm}}^{f\kern0.1em g}\equiv \left\langle {\varphi}_{f\mathbf{n}}\left(\mathbf{r}\right)\right|\;W\left(\mathbf{r}\right)-\left(1/13\right){\displaystyle \sum_{\mathbf{l}}\left(z{e}^2/\left|\mathbf{r}-\mathbf{l}\right|\right)}\;\left|{\varphi}_{g\;\mathbf{m}}\left(\mathbf{r}\right)\right\rangle$ is the matrix element of the difference of interaction energies of the electron with external influences. In this case, *W*(**r**) is the external field, while $\left(1/13\right){\displaystyle \sum_{\mathbf{l}}\left(z{e}^2/\left|\mathbf{r}-\mathbf{l}\right|\right)}$ [14] is the effective external field, which is associated with the amino acid inhomogeneity. As it may be inferred from this definition, under certain circumstances $\left(\mathrm{f}\mathrm{o}\mathrm{r}\ \mathrm{example},\ W\left(\mathbf{r}\right)\approx \left(1/13\right){\displaystyle \sum_{\mathbf{l}}\left(z{e}^2/\left|\mathbf{r}-\mathbf{l}\right|\right)}\right)$, this energy may be a negligible quantity.

Operators of occupation and operators of escapement (creation/annihilation operators) of the electronic states ${b}_{f\mathbf{n}}^{+}$, *b*_*f***n**_ satisfy the anticommutation relations:

2 $$ {b}_{f\mathbf{n}}{b}_{g\kern0.1em \mathbf{m}}^{+}+{b}_{g\kern0.1em \mathbf{m}}^{+}{b}_{f\mathbf{n}}={\delta}_{fg}{\delta}_{\mathbf{nm}}\kern0.5em ;\kern0.5em {b}_{f\mathbf{n}}{b}_{g\kern0.1em \mathbf{m}}+{b}_{g\kern0.1em \mathbf{m}}{b}_{f\mathbf{n}}=0\kern0.5em ;\kern0.5em {b}_{f\mathbf{n}}^{+}{b}_{g\kern0.1em \mathbf{m}}^{+}+{b}_{g\kern0.1em \mathbf{m}}^{+}{b}_{f\mathbf{n}}^{+}=0 $$

and act upon the functions of the filling numbers | … , *N*_*f***n**_, … 〉. Variables within these functions *N*_*f***n**_ will take only two values: *N*_*f***n**_ = 0, if the state is not filled-in, and *N*_*f***n**_ = 1, if the state is filled-in. Influence of the operators ${b}_{f\mathbf{n}}^{+}$, *b*_*f***n**_ upon the functions of the filling numbers is determined by the following relationships:

$$ {b}_{f\mathbf{n}}^{+}\left| \dots, {N}_{f\mathbf{n}},\dots \right\rangle ={\left(-1\right)}^{\chi_{f\mathbf{n}}}\left(1-{N}_{f\mathbf{n}}\right)\left| \dots, 1-{N}_{f\mathbf{n}},\dots \right\rangle; \kern0.5em {b}_{f\mathbf{n}}\left| \dots, {N}_{f\mathbf{n}},\dots \right\rangle ={\left(-1\right)}^{\chi_{f\mathbf{n}}}{N}_{f\mathbf{n}}\left| \dots, 1-{N}_{f\mathbf{n}},\dots \right\rangle $$

Index of power *χ*_*f***n**_ is equal to the quantity of the filled-in states, which are situated before the state *f***n**.

State of the system under consideration (protein nanowire—electron, which was injected into the conductivity band *f* = *с*) may be described with the help of the wave function as follows:

3 $$ \left|1\right\rangle ={\displaystyle \sum_{\mathbf{n}}{a}_{\mathbf{n}}{b}_{c\kern0.1em \mathbf{n}}^{+}\left|0\right\rangle } $$

Vacuum state |0〉 of the entire system is determined in Eq. (3) in such a manner that filling numbers for all valence bands (*E*_*f*_ < *E*_*c*_) at the zero temperature are equal to 1, while for all conductivity bands filling numbers are equal to 0. With the help of the anticommutation relations (2), it is not difficult to show that operation 〈1| 1〉 will be reduced to the equation: $\left\langle 1|1\right\rangle ={\displaystyle \sum_{\mathbf{n}}{\left|{a}_{\mathbf{n}}\right|}^2}$. This is the basis for formulation of the normalisation condition 〈1| 1〉 = 1. With the help of this condition, it is possible to attach probabilistic meanings to the values |*a*_**n**_|^2^, as well as attach meanings of the wave functions of the variable **n** to the values *a*_**n**_. Orthogonality conditions are met as well: 〈1| 0〉 = 〈0| 1〉 = 0. With the help of averaging the operator (1) over functions (3), that is, due to execution of the operation *E*({*a*}) = 〈1|*Ĥ*|1〉, we will find the efficient functional in respect of the functions *a*_**n**_:

4 $$ E\left(\left\{a\right\}\right)={\displaystyle \sum_{\mathbf{n}\;\mathbf{m}}{}^{/}w_{\mathbf{n}\;\mathbf{m}}}+{\displaystyle \sum_{\mathbf{n}}\left({\tilde{W}}_{\mathbf{n}\kern0.1em \mathbf{n}}^{\kern0.1em c\kern0.1em c}+{D}_{\mathbf{n}}^c\right)\cdot {\left|{a}_{\mathbf{n}}\right|}^2}+2{\displaystyle \sum_{\mathbf{n}\;\mathbf{m}}{}^{/}M_{\mathbf{n}\kern0.1em \mathbf{m}}^{c\kern0.5em c}\;{a}_{\mathbf{n}}^{\ast }{a}_{\mathbf{m}}} $$

In accordance with the above-presented meanings of the matrix elements ${Q}_{\mathbf{n}\kern0.1em \mathbf{l}\kern0.1em \mathbf{m}}^{\kern0.1em fg}$, ${V}_{\mathbf{n}\mathbf{m}{\mathbf{n}}^{\mathbf{\prime}}{\mathbf{m}}^{\mathbf{\prime}}}^{fg\kern0.1em {f}^{\prime}\kern0.1em {g}^{\prime }}$, and ${\tilde{W}}_{\mathbf{nm}}^{f\kern0.1em g}$, the following values were introduced in the functional (4) for the energy of the protein molecule, which was excited as the result of the injection of an electron. ${w}_{\mathbf{n}\kern0.1em \mathbf{m}}\equiv {K}_{\mathbf{n}-\mathbf{m}}/2-{\displaystyle \sum_f\left[{N}_{f\mathbf{0}}^{(T)}{Q}_{\mathbf{0},\mathbf{m}-\mathbf{n},\mathbf{0}}^{\kern1em f\kern1em f}-{\displaystyle \sum_g{N}_{f\mathbf{0}}^{(T)}{N}_{g\kern0.1em \mathbf{0}}^{(T)}{\gamma}_{\mathbf{n}\kern0.1em \mathbf{m}\kern0.1em \mathbf{m}\kern0.1em \mathbf{n}}^{fg\kern0.2em g\kern0.1em f}/2}\right]}$ is the energy of the interatomic interaction. This interaction plays the leading part in the course of consolidation of the isolated atoms into the single-coupled system. In this case, additional values and meanings are introduced in order to simplify entries and records: *K*_**n** − **m**_ = (*ze*)^2^/|**n** − **m**| is the direct Coulomb interaction between atomic nuclei and ${\gamma}_{\mathbf{n}\;\mathbf{m}\;{\mathbf{n}}^{\mathbf{\prime}}{\mathbf{m}}^{\mathbf{\prime}}}^{fg\;{f}^{\prime}\;{g}^{\prime }}\equiv {V}_{\mathbf{n}\;\mathbf{m}\;{\mathbf{n}}^{\mathbf{\prime}}{\mathbf{m}}^{\mathbf{\prime}}}^{fg\;{f}^{\prime}\;{g}^{\prime }}-{V}_{\mathbf{n}\;\mathbf{m}\;{\mathbf{m}}^{\mathbf{\prime}}{\mathbf{n}}^{\mathbf{\prime}}}^{fg\;{g}^{\prime}\kern0.5em {f}^{\prime }}$. Factors of the type ${N}_{f\mathbf{0}}^{(T)}$ are in fact Fermi-Dirac distributions. As concerns temperature, these distributions have the following form with sufficient degree of accuracy: ${N}_{f\kern0.1em \mathbf{0}}^{(T)}={\left[1+ \exp \left\{\left({E}_f-\mu \right)/kT\right\}\right]}^{-1}$, where *μ* is the chemical potential. In the conditions under consideration, *μ* is determined by the following relationship: *μ* = (*E*_*с*_ + *E*_*ν*_)/2, where “*c*” subscript is used in order to denote the conductivity band, which is characterised by the lowest energy, while “*v*” subscript denotes the valence band, which is the closest one to the above-mentioned conductivity band. As concerns energies of *E*_*f*_ type in a certain approximation, in accordance with which it is possible to neglect the details concerning influence of the temperature, it may be worth to consider the following relationship: ${E}_f\equiv {N}_a\left({\varepsilon}_f+{\displaystyle \sum_g{\gamma}_{\mathbf{0}\kern0.5em \mathbf{0}\kern0.5em \mathbf{0}\;\mathbf{0}}^{fg\;gf}/2}\right)$. However, in the cases where these details are essential ones, then: ${E}_f\equiv {N}_a\left({N}_{f\mathbf{0}}^{(T)}{\varepsilon}_f+{\displaystyle \sum_g{N}_{f\mathbf{0}}^{(T)}{N}_{g\kern0.1em \mathbf{0}}^{(T)}{\gamma}_{\mathbf{0}\kern0.5em \mathbf{0}\kern0.5em \mathbf{0}\;\mathbf{0}}^{fg\;gf}/2}\right)$. In the absence of the electron injection, the equilibrium-coupled state of the atomic system is determined by the minimum of energy *w*_**n m**_ over the vector components **n** − **m**. ${D}_{\mathbf{n}}^c\equiv -{\displaystyle \sum_{\mathbf{m}\left(\ne \mathbf{n}\right)}\left[{Q}_{\mathbf{0},\mathbf{m}-\mathbf{n},\mathbf{0}}^{\kern0.6em c\kern.72em c}-{\displaystyle \sum_f{N}_{f\mathbf{0}}^{(T)}{\gamma}_{\mathbf{n}\kern0.1em \mathbf{m}\kern0.1em \mathbf{m}\kern0.1em \mathbf{n}}^{c\kern0.5em f\kern0.1em f\kern0.5em c}}\right]}$ is an additional energy to *w*_**n m**_. This energy, as it may be seen from the structure of the functional (4), is substantially connected with the excitation and it disturbs the equilibrium state of the system. Such disturbance causes occurrence of nonlinearity in the system at the expense of the self-influence of the excitation due to the response of the crystal lattice to such excitation. ${\tilde{W}}_{\mathbf{n}\kern0.1em \mathbf{n}}^{\kern0.1em c\kern0.1em c}\equiv \left\langle {\varphi}_{c\;\mathbf{n}}\left(\mathbf{r}\right)\right|\;W\left(\mathbf{r}\right)-\left(1/13\right){\displaystyle \sum_{\mathbf{l}}\left(z{e}^2/\left|\mathbf{r}-\mathbf{l}\right|\right)}\;\left|{\varphi}_{c\;\mathbf{n}}\left(\mathbf{r}\right)\right\rangle$ represents influence of the external fields (both actual and effective ones) upon the injected electron. In the case of availability, only actual external field, energy of s electron may be represented for the most part with the help of the following relationship: *W*(**r**) = (**r** ⋅ **J**), where **J** − is the vector force constant (in respect of the variable **r**). Then:

$$ {W}_{\mathbf{n}\kern0.1em \mathbf{n}}^{\kern0.1em c\kern0.1em c}=\left\langle {\varphi}_c\left(\mathbf{r}-\mathbf{n}\right)\kern0.24em \left|\left(\mathbf{r}\cdot \mathbf{J}\right)\right|{\varphi}_c\left(\mathbf{r}-\mathbf{n}\right)\right\rangle =\left\langle {\varphi}_c\left(\boldsymbol{\uprho} \right)\kern0.24em \left|\left(\left(\boldsymbol{\uprho} +\mathbf{n}\right)\cdot \mathbf{J}\right)\right|{\varphi}_c\left(\boldsymbol{\uprho} \right)\right\rangle =\left({\boldsymbol{\uprho}}_{c\kern0.1em c}\cdot \mathbf{J}\right)+\left(\mathbf{n}\cdot \mathbf{J}\right) $$

As concerns the above-shown basis in the form of wave functions of the single-electronic ion, the following condition is always met: **ρ**_*с с*_ = **0**. Then the considered energy of the external field is reduced to the following form: ${W}_{\mathbf{n}\kern0.1em \mathbf{n}}^{\kern0.1em c\kern0.1em c}=\left(\mathbf{n}\cdot \mathbf{J}\right)$. At last, the matrix element ${M}_{\mathbf{n}\kern0.1em \mathbf{m}}^{c\kern0.5em c}$, which is included to the functional (4), is determined with the help of the strict equality: ${M}_{\mathbf{n}\kern0.1em \mathbf{m}}^{c\kern0.5em c}\equiv -\left(1/2\right){\displaystyle \sum_{\mathbf{l}}\left[{Q}_{\mathbf{0},\mathbf{l}-\mathbf{n},\mathbf{m}-\mathbf{n}}^{\kern0.6em c\kern1.5em c}-{\displaystyle \sum_f{N}_{f\mathbf{0}}^{(T)}{\gamma}_{\mathbf{n}\kern0.22em \mathbf{l}\kern0.22em \mathbf{l}\kern0.22em \mathbf{m}}^{c\;f\kern0.1em f\;c}}\right]}$. It is an additional summand to the energy (in the same manner as ${D}_{\mathbf{n}}^c$) and describes dynamics of excitation, if it would be considered as a quasi-particle, that is, as the object of a classic type. That is, the matrix element ${M}_{\mathbf{n}\kern0.1em \mathbf{m}}^{c\kern0.5em c}$, being the part of energy of the system, is the actual value and it satisfies the following condition: ${M}_{\mathbf{n}\kern0.1em \mathbf{m}}^{c\kern0.5em c}={M}_{\mathbf{m}\kern0.1em \mathbf{n}}^{c\kern0.5em c}$. Then it is possible to reduce functional (4) to the following final form:

5 $$ E\left(\left\{a\right\}\right)={\displaystyle \sum_{\mathbf{n}\;\mathbf{m}}{}^{/}w_{\mathbf{n}\;\mathbf{m}}}+{\displaystyle \sum_{\mathbf{n}}\left({\tilde{W}}_{\mathbf{n}\kern0.1em \mathbf{n}}^{\kern0.1em c\kern0.1em c}+{D}_{\mathbf{n}}^c\right)\cdot {\left|{a}_{\mathbf{n}}\right|}^2}+{\displaystyle \sum_{\mathbf{n}\;\mathbf{m}}{}^{/}M_{\mathbf{n}\kern0.1em \mathbf{m}}^{c\kern0.5em c}\;\left({a}_{\mathbf{n}}^{\ast }{a}_{\mathbf{m}}+{a}_{\mathbf{m}}^{\ast }{a}_{\mathbf{n}}\right)} $$

Such detailed meanings of the values, which are included to the functional (5), are required in order to have further possibilities for analysis of the issues under consideration not only at the quality level, but in order to make adequate quantitative estimates (which are important ones for the diagnostic and treatment understanding) as well.

## Results and Discussion

### States of Conductivity of the Protein Molecule

Regardless of the fact that the primary structure of the protein molecule has a spiral form (α-spiral), we will analyse this structure as a linear one. This is explained by the fact that taking into account the spirality of its form is only important in the cases where direction of the external electric field does not coincide with the axis of the α-spiral. Then, the injected electron is in the conditions of the spatial-periodical field, and this problem will require a special analysis and discussion. In this case, the question is connected with calculation of the conductivity states, upon which configuration of the protein nanowire in the conditions of absence of external fields has only a slight impact. Therefore, we will restrict our analysis with linear structure in order to simplify the discussion.

In order to ensure approximation of the linearly elongated protein nanowire, spatial variables **n**, **m** in the functional (5) will loose their vector nature, and functional (5) will have the following form:

6 $$ E\left(\left\{a\right\}\right)={\displaystyle {\sum_{n\kern0.1em m}}^{/}{w}_{n\;m}}+{\displaystyle \sum_n\left({\tilde{W}}_{n\;n}^{\;c\kern0.1em c}+{\displaystyle \sum_{m\left(\ne n\right)}{D}_{n\;m}^{\kern0.1em c\;c}}\right)}{\left|{a}_n\right|}^2+{\displaystyle {\sum_{n\kern0.1em m}}^{/}{M}_{n\;m}^{\kern0.1em c\;c}\left({a}_n^{\ast }{a}_m+{a}_m^{\ast }{a}_n\right)} $$

In addition, the above equation in this case takes into account the following notion: ${D}_{\mathbf{n}}^c\equiv -{\displaystyle \sum_{\mathbf{m}\left(\ne \mathbf{n}\right)}\left[{Q}_{\mathbf{0},\mathbf{m}-\mathbf{n},\mathbf{0}}^{\kern0.6em c\kern1.72em c}-{\displaystyle \sum_f{N}_{f\mathbf{0}}^{(T)}{\gamma}_{\mathbf{n}\kern0.1em \mathbf{m}\kern0.1em \mathbf{m}\kern0.1em \mathbf{n}}^{c\kern0.5em f\kern0.1em f\kern0.5em c}}\right]}$, from which it is possible to obtain an expression: ${D}_{\mathbf{n}\mathbf{m}}^{\kern0.1em c\kern0.1em c}\equiv -\left({Q}_{\mathbf{0},\mathbf{m}-\mathbf{n},\mathbf{0}}^{\kern0.6em c\kern.72em c}-{\displaystyle \sum_f{N}_{f\mathbf{0}}^{(T)}{\gamma}_{\mathbf{n}\kern0.1em \mathbf{m}\kern0.1em \mathbf{m}\kern0.1em \mathbf{n}}^{c\kern0.5em f\kern0.1em f\kern0.5em c}}\right)$, and for the spatially one-dimensional situation, which is under consideration, the expression will be as follows: ${D}_{n\;m}^{\kern0.1em c\;c}=-\left({Q}_{0,m-n,0}^{\kern0.6em c\kern.72em c}-{\displaystyle \sum_f{N}_{f0}^{(T)}{\gamma}_{n\kern0.1em m\kern0.1em m\kern0.1em n}^{c\kern0.5em f\kern0.1em f\;c}}\right)$.

Now, we will use the approximation of the nearest neighbours, which is typical for the one-dimensional molecular structures, when all double amounts of the functional (6) will only take into account the summands, which correspond to the neighbouring atoms: *m* = *n* + *R*_*n*_, where *R*_*n*_ is the distance between neighbouring atoms (this distance may be different for different values of *n*).

**Fig. 1:**
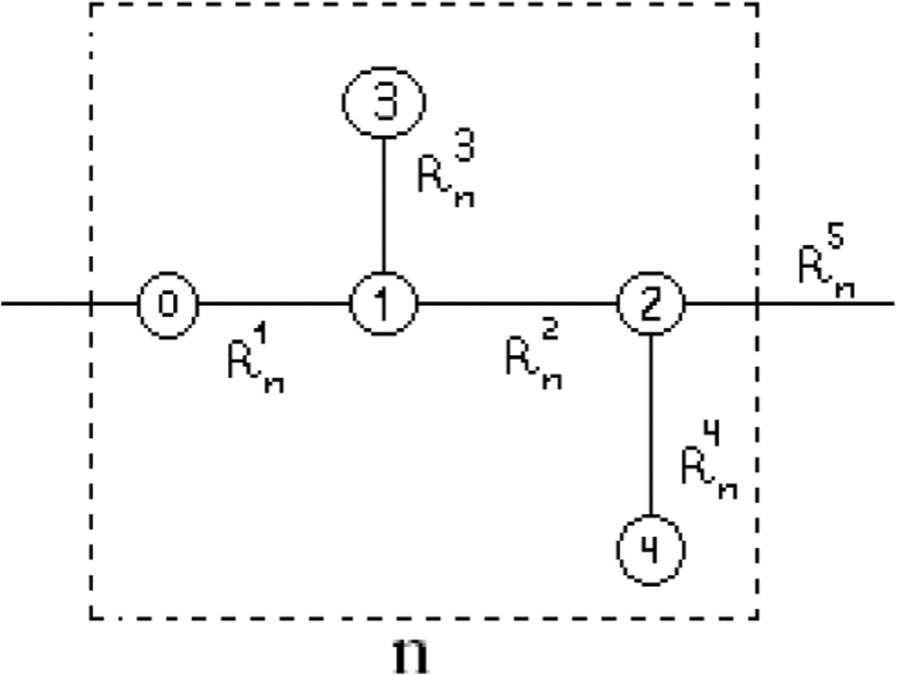
Schematic presentation of the amino acid residual. *Dash lines* represent its conventional borders. *Digits* represent quantities of atoms within each such group. Within the nitrogen model of the nuclear subsystem, all atoms are similar; ${R}_{\kern.34em n}^{\alpha +1}-$ distances between atoms (*α* = 0, …, 4)

If we will analyse the structure of the periodically recurring molecular group in the proteins (in fact, it is the structure of the averaged amino acid residual) [14], then, in accordance with the nitrogen model of the nuclear subsystem, the spatial variable *n* will not determine a separate atom. In this case, it will determine the entire amino acid residual already. Therefore, it would be necessary to introduce the second subscript for the atoms that are present within this residual, for example, α, which will include all atoms at the fixed value of *n*. Therefore, periodically recurring molecular groups will have the form, which is schematically presented in Fig. 1 for the certain *n*th amino acid residual in the protein nanowire.

If we will know the spatial organisation of the elementary cell, it will be possible to reproduce a relevant subzone structure of the conductivity band. In accordance with Fig. 1, all matrix elements now will be numbered with the help of the double subscript *n*, *α*, where *α* = 0, 1, 2, 3, 4, while *n*  =  1,  …, *N*_0_, where *N*_0_ is the quantity of the amino acid residuals, but it is not the quantity of separate atoms, as it was earlier in the functionals (4)–(6).

In the presence of an excitation in the form of the electron, which was injected into the conductivity band, interatomic equilibrium disturbs in such a manner that equilibrium distances ${R}_{\kern.5em n}^{\alpha +1}$ change quite significantly and such excitation takes form of a soliton wave. For the time being, we will neglect this effect in order to pay attention to the conductivity states.

Let us consider that the equilibrium state is not disturbed, as well as that conditions ${R}_{\kern.5em n}^{\alpha +1}={R}_{\alpha +1}\equiv {R}_0$ are met. Then, with the help of the transformation of the functional (6) in accordance with the latter supposition, as well as in accordance with Fig. 1, we may determine such working form of this functional:

7 $$ \begin{array}{c}E\left(\left\{a\right\}\right)={N}_aw+{\displaystyle \sum_{n=1}^{N_0}\left[{\displaystyle \sum_{\alpha =0}^4\left({\tilde{W}}_n^{\alpha }+{P}_{\alpha}\right){\left|{a}_{n\kern0.1em \alpha}\right|}^2+}\right.}M\left\{{\displaystyle \sum_{\alpha =0}^1\left({a}_{n\kern0.1em \alpha}^{*}{a}_{n,\;\alpha +1}+{a}_{n,\;\alpha +1}^{*}{a}_{n\kern0.1em \alpha}\right)+}\right.\\ {}\left.\left.+{\displaystyle \sum_{\alpha =2}^3\left({a}_{n,\;\alpha -1}^{*}{a}_{n,\;\alpha +1}+{a}_{n,\;\alpha +1}^{*}{a}_{n,\;\alpha -1}\right)+}\left({a}_{n\kern0.1em 2}^{*}{a}_{n+1,\;0}+{a}_{n+1,\;0}^{*}{a}_{n\kern0.1em 2}\right)\right\}\right]\kern0.5em .\end{array} $$

Within this functional, “*c*” subscripts, which symbolise conductivity band, are eliminated. *N*_*a*_ is the quantity of atoms, while *N*_0_ = *N*_*a*_/5 is the quantity of the amino acid residuals. Designations for *w*≡*w*(*R*_0_) and *M*≡*M*(*R*_0_) may be obtained in a successive order from the general specifications:

8 $$ {w}_{\mathbf{n}\kern0.1em \mathbf{m}}\equiv {K}_{\mathbf{n}-\mathbf{m}}/2-{\displaystyle \sum_f\left[{N}_{f\mathbf{0}}^{(T)}{Q}_{\mathbf{0},\mathbf{m}-\mathbf{n},\mathbf{0}}^{\kern1em f\kern1em f}-{\displaystyle \sum_g{N}_{f\mathbf{0}}^{(T)}{N}_{g\kern0.1em \mathbf{0}}^{(T)}{\gamma}_{\mathbf{n}\kern0.1em \mathbf{m}\kern0.1em \mathbf{m}\kern0.1em \mathbf{n}}^{fg\kern0.2em g\kern0.1em f}/2}\right]} $$

9 $$ {M}_{\mathbf{n}\kern0.1em \mathbf{m}}^{c\kern0.5em c}\equiv -\left(1/2\right){\displaystyle \sum_{\mathbf{l}}\left[{Q}_{\mathbf{0},\mathbf{l}-\mathbf{n},\mathbf{m}-\mathbf{n}}^{\kern0.6em c\kern.5em c}-{\displaystyle \sum_f{N}_{f\mathbf{0}}^{(T)}{\gamma}_{\mathbf{n}\kern0.22em \mathbf{l}\kern0.22em \mathbf{l}\kern0.22em \mathbf{m}}^{c\;f\kern0.1em f\;c}}\right]} $$

Field summand ${\tilde{W}}_n^{\alpha }$ is denoted as follows: ${\tilde{W}}_n^{\alpha}\equiv {\tilde{W}}_{n\kern0.1em \alpha,\;n\kern0.1em \alpha}^{\kern0.5em c\;c}$, while coefficients *P*_*α*_ taking into account designation *D*≡*D*^*с с*^(*R*_0_), as well as the general specification:

10 $$ {D}_{n\;m}^{\kern0.1em c\;c}={Q}_{0,m-n,0}^{\kern0.6em c\kern.72em c}-{\displaystyle \sum_f{N}_{f0}^{(T)}{\gamma}_{n\kern0.1em m\kern0.1em m\kern0.1em n}^{c\kern0.5em f\kern0.1em f\;c}} $$

are determined by the relationships: ${P}_0\equiv {D}^{c\kern0.1em c}\left({R}_n^1\right)\equiv D$; ${P}_1\equiv {D}^{cc}\left({R}_n^2\right)+{D}^{cc}\left({R}_n^3\right)/2\equiv 3D/2$; ${P}_2\equiv {D}^{c\kern0.1em c}\left({R}_n^4\right)/2+{D}^{c\kern0.1em c}\left({R}_n^5\right)\equiv 3D/2$; ${P}_3\equiv {D}^{c\kern0.1em c}\left({R}_n^3\right)/2\equiv D/2$; and ${P}_4\equiv {D}^{c\kern0.1em c}\left({R}_n^4\right)/2\equiv D/2$. Now, we will perform variation of the conventional functional of the considered system: ${E}_{\mathrm{cond}}\left(\left\{a\right\}\right)=E\left(\left\{a\right\}\right)+\varepsilon \left(1-{\displaystyle \sum_{n=1}^{N_0}{\displaystyle \sum_{\alpha =0}^4{\left|{a}_{n\kern0.1em \alpha}\right|}^2}}\right)$, where the main part of this functional is determined in (7), while its conventional part is connected with the normalisation condition: ${\displaystyle \sum_{n=1}^{N_0}{\displaystyle \sum_{\alpha =0}^4{\left|{a}_{n\kern0.1em \alpha}\right|}^2}}=1$. Parameter *ε* is the energy eigenvalue. As the result of variation of the functional *E*_cond_({*a*}) in respect of the functions *a*_*n α*_ on the condition of ${\tilde{W}}_n^{\alpha }=0$, as well as due to presentation of these functions in the form of: *a*_*n α*_ = A_*α*_ exp(*i kR*_0_*n*), it is possible to obtain such system consisting of five equations in order to determine both A_*α*_ coefficients, and *x*≡*ε*/|*D*| eigenvalue, which determine energy *ε*:

$$ \left(x+1\right){\mathrm{A}}_0+v{\mathrm{A}}_1+v{e}^{-ik{R}_0}{\mathrm{A}}_2=0;\kern0.5em v{\mathrm{A}}_0+\left(x+3/2\right){\mathrm{A}}_1+v{\mathrm{A}}_2+v{\mathrm{A}}_3=0;\kern0.5em v{e}^{ik{R}_0}{\mathrm{A}}_0+v{\mathrm{A}}_1+\left(x+3/2\right){\mathrm{A}}_2+v{\mathrm{A}}_4=0;\kern0.5em v{\mathrm{A}}_1+\left(x+1/2\right){\mathrm{A}}_3=0;\kern0.5em v{\mathrm{A}}_2+\left(x+1/2\right){\mathrm{A}}_4=0 $$

In this case, we have introduced new designation: *v*≡|*M*/*D*|, as well as we have used Born-Karman condition: ${a}_{1\kern0.5em \alpha }={a}_{N_0+1\kern0.5em \alpha }$, which determines the wave vector: *k*  = 2*π j*/*N*_0_*R*_0_, where, as usual, *j* will take integer values: *j* = 0, ± 1, ± 2, …, of which only the first *N*_0_ values correspond to the independent solutions.

Compatibility condition of this system leads to the equation of the fifth degree in respect of the non-dimensional eigenvalue *x*:

11 $$ \begin{array}{c}{x}^5+5{x}^4+\frac{1}{2}\left(19-10{v}^2\right){x}^3+\frac{1}{2}\left(17-26{v}^2+4{v}^3 \cos \left(k{R}_0\right)\right){x}^2+\\ {}+\frac{1}{16}\left(57-164{v}^2+32{v}^3 \cos \left(k{R}_0\right)+48{v}^4\right)x+\frac{1}{16}\left(9-40{v}^2+8{v}^3 \cos \left(k{R}_0\right)+32{v}^4\right)=0\kern0.5em .\end{array} $$

The detailed graphical and numerical analysis of Eq. (11) makes it possible to develop the approximation solution of this equation. Because of there exist five such solutions, it is suitable to present the same in the matrix form and describe all five roots of Eq. (11) with the help of one relationship:

12 $$ {x}_s\left(v,k\right)=-\left(\begin{array}{c}\hfill 1.0\hfill \\ {}\hfill \begin{array}{l}3/2\\ {}3/2\\ {}1/2\\ {}1/2\end{array}\hfill \end{array}\right)+\kern0.5em \left(\begin{array}{c}\hfill\;{v}^2\hfill \\ {}\hfill \begin{array}{l}\kern1em v\\ {}-v\\ {}\kern1em {v}^2\\ {}\kern1em {v}^2\end{array}\hfill \end{array}\right)+\kern0.5em \left(\begin{array}{c}\hfill \kern0.5em 3.15\;{v}^4\hfill \\ {}\hfill \begin{array}{l}-2.49{v}^2\\ {}-1.42{v}^2\\ {}-1.23{v}^4\\ {}\kern1em 3.72{v}^4\end{array}\hfill \end{array}\right)-{v}^3\left(\begin{array}{c}\hfill \kern0.5em 0.68\hfill \\ {}\hfill \begin{array}{l}-3.10\\ {}\kern1em 1.90\\ {}-0.35\\ {}\kern1em 0.87\end{array}\hfill \end{array}\right) \cos \left(k{R}_0\right)\kern0.5em . $$

State subscript “*s*” will take five values (for example, from 0 to 4) and within the representation (12), it will accept these solutions from the top to the bottom. In spite of the approximate nature of these solutions, they are enough accurate as concerns quantitative understanding practically for any values within the interval 0 < *v* < 1, more particularly for the values that are close to *v* = 0.4. In the case of deviation from the value *v* = 0.4, numerical factors in the third summand of the right part of the expression (12) may slightly change. From the physical point of view, relationship of the resonance exchange energy |*M*| to the main part of the excitation energy |*D*|: *v*≡|*M*/*D*| will ensure fulfilment of the following condition: 0 < *v* < 1.

It is obvious that at *v* ≠ 0, all eigenvalues are not degenerate values. Only in the idealised conditions, at *v* = 0, we will observe a pairwise degeneracy of two states: *x*_1_(*v*, *k*) = *x*_2_(*v*, *k*) and *x*_3_(*v*, *k*) = *x*_4_(*v*, *k*). In this case, one pair of these states *x*_1,2_(*v*, *k*) is always situated energetically lower lying of the non-degenerate state *x*_0_(*v*, *k*), while the second pair *x*_3,4_(*v*, *k*) is always situated energetically higher lying of the non-degenerate state *x*_0_(*v*, *k*). As concerns each root of Eq. (12), if we will substitute this root into the output system, we will obtain

$$ \left(x+1\right){\mathrm{A}}_0+v{\mathrm{A}}_1+v{e}^{-ik{R}_0}{\mathrm{A}}_2=0;\kern0.5em v{\mathrm{A}}_0+\left(x+3/2\right){\mathrm{A}}_1+v{\mathrm{A}}_2+v{\mathrm{A}}_3=0;\kern0.5em v{e}^{ik{R}_0}{\mathrm{A}}_0+v{\mathrm{A}}_1+\left(x+3/2\right){\mathrm{A}}_2+v{\mathrm{A}}_4=0;\kern0.5em v{\mathrm{A}}_1+\left(x+1/2\right){\mathrm{A}}_3=0;\kern0.5em v{\mathrm{A}}_2+\left(x+1/2\right){\mathrm{A}}_4=0 $$

At the same time, taking into account the normalisation condition: ${\displaystyle \sum_{n=1}^{N_0}{\displaystyle \sum_{\alpha =0}^4{\left|{a}_{n\kern0.1em \alpha}\right|}^2}}=1$, it is possible to find five solutions for A_*α*_ coefficients in the form of five sets for them. However, there is no necessity to do so in the conditions of the absence of external fields. One thing is only important: it is necessary to state the fact of existence of these solutions, as well as the fact that they are normalised to the figure of one.

With the help of representation (12) and designation *x*≡*ε*/|*D*|, it is possible to find out actual (dimensional) eigenvalues of energy: *ε*_*s*_(*v*, *k*) = |*D*| *x*_*s*_(*v*, *k*).

In order to formulate the final determination for the energy of the “protein nanowire-injected electron” system (this energy will be an important figure later on), we will return to the conventional functional: ${E}_{\mathrm{cond}}\left(\left\{a\right\}\right)=E\left(\left\{a\right\}\right)+\varepsilon \left(1-{\displaystyle \sum_{n=1}^{N_0}{\displaystyle \sum_{\alpha =0}^4{\left|{a}_{n\kern0.1em \alpha}\right|}^2}}\right)$. On the one hand, the second summand (along with the multiplicand *ε*) within this expression for the energy is identically transformed into 0, if *a*_*n α*_ = A_*α*_ exp(*i kR*_0_*n*) solutions exist and if they are normalised. Then, the following relationship: *E*_cond_({*a*}) = *E*({*a*}) is obvious. On the other hand, in order to clear up the question on the explicit form of energy *E*({*a*}), as well as on the explicit form of energy *E*_cond_({*a*}) (without necessity to find out the explicit forms of the wave functions *a*_*n α*_ = A_*α*_ exp(*i kR*_0_*n*)), we will utilise the following considerations. This same energy may be described in the form as follows: ${E}_{\mathrm{cond}}\left(\left\{a\right\}\right)=\varepsilon +E\left(\left\{a\right\}\right)-\varepsilon {\displaystyle \sum_{n=1}^{N_0}{\displaystyle \sum_{\alpha =0}^4{\left|{a}_{n\;\alpha}\right|}^2}}$. The eigenfunctions *a*_*n α*_ = A_*α*_ exp(*i kR*_0_*n*), if they were found, will ensure transformation of $E\left(\left\{a\right\}\right)-\varepsilon {\displaystyle \sum_{n=1}^{N_0}{\displaystyle \sum_{\alpha =0}^4{\left|{a}_{n\;\alpha}\right|}^2}}$ (the part of the difference, which depends on *a*_*nα*_) into 0 (zero). It follows from the expression (7), that only product *N*_*a*_*w* will remain from this difference, and at the same time, we will have the following expression for the energy, which we want to determine: *E*_cond_({*a*}) = *N*_*a*_*w* + *ε*. Therefore, taking into account the equality: *E*_cond_({*a*}) = *E*({*a*}), we at last will come to the following expression:

$$ {E}_s\left(v,k\right)={N}_aw+{\varepsilon}_s\left(v,k\right)\equiv {N}_aw+\left|D\right|\kern0.5em {x}_s\left(v,k\right) $$

The energy *E*_*s*_(*v*, *k*) may be detailed up to the calculated-and-evaluative level with the help of expressions (8)–(10). Particularly, such detalization may be interested from the special point of view, where it may help to determine influence of the temperature upon the electron subsystem, as well as upon the microcurrent due to the presence of the injected electron in the conditions of availability of external fields. However, we will not detail this energy here, because the main goal of this research is not connected with the detailed calculation of the determined energies; this research is aimed at the qualitative analysis of the general properties of conductivity.

### Determination of Current Density

Current density has such a general definition: **j** = *e n***V**, where *e* is the charge of an electron; *n* is the average volume density of charges. As concerns a single-injected electron, this density (with the accuracy to the *e n* product, which is a constant value for each protein molecule) in fact is determined by the velocity **V**. Taking into consideration the fact that current is to be determined based on the consideration on an injected electron, it is necessary to consider this electron as a free quasi-particle of the classic type within the conductivity band of the primary structure of the protein molecule [19–21]. In this case, each of the eigenvalues *E*_*s*_(*v*, *k*) for the energies of subzones is to be considered as a classic Hamiltonian of the wave pulse *p* = *ℏ k* [21, 22].

The above was a brief justification of the possibility of review of the primary structure of the protein molecule as a linear elongated object. Therefore, taking into consideration the Hamiltonian pattern of energy *E*_*s*_(*v*, *k*) in respect of the wave vector *k*, it would be sufficient to analyse only the value of velocity in the spatially one-dimensional situation (this velocity is a standard one for the solid bodies): V_*s*_(*v*, *k*) = (1/*ℏ*)[*dE*_*s*_(*v*, *k*)/*dk*]. It is important to underline that negativity of the value V_*s*_(*v*, *k*) is to be interpreted as the opposite directionality of the velocity vector in respect of those direction, which was determined as the positive one.

With the help of the explicit form of the energy *E*_*s*_(*v*, *k*) in the expression for the velocity, it may be found out that: $$ {\mathrm{V}}_s\left(v,k\right)=\frac{1}{\hslash}\left|D\right|{R}_0\left(\begin{array}{c}\hfill \kern0.5em 0.68\hfill \\ {}\hfill \begin{array}{l}-3.10\\ {}\kern1em 1.90\\ {}-0.35\\ {}\kern1em 0.87\end{array}\hfill \end{array}\right){v}^3 \sin \left(k{R}_0\right) $$. As it may be inferred from this expression, two of these velocities (the velocities, which correspond to the “abnormal” subzones *E*_1_(*v*, *k*) and *E*_3_(*v*, *k*)) are presented as negative values as compared with the other (“normal”) subzones. Therefore, in accordance with the general definition **j** = *e* n **V**, we will have for the current densities the following expression: $$ {\mathrm{j}}_s=\frac{e\left|D\right|{R}_0}{\hslash {N}_0{V}_0}\left(\begin{array}{c}\hfill \kern0.5em 0.68\hfill \\ {}\hfill \begin{array}{l}-3.10\\ {}\kern1em 1.90\\ {}-0.35\\ {}\kern1em 0.87\end{array}\hfill \end{array}\right){v}^3 \sin \left(k{R}_0\right) $$. Here, we have taken into account that for a single-injected electron: n ≡1/*N*_0_*V*_0_, where *V*_0_ is the effective average volume of the amino acid residual, while *N*_0_ is the quantity of the amino acid residuals (as it was already noted above).

Based on any physical considerations (both classic ones, and quantum ones; “classic language” says about parallel connection, while “quantum language” says about equal probability of the conductivity channels), it is obvious that the value of the general current density *j* within the entire conductivity band is determined by the following sum: $j={\displaystyle \sum_{s=0}^4{j}_s}$. If we will substitute the values *j*_*s*_, which we have just determined, we will obtain: *j* = 0. This result (which is an unexpected one, at the first glance) we have to consider in effect as the test result concerning correctness of the entire previous analysis. Because of the conditions where there are no any factors, which would disturb an electrostatic equilibrium of the system and which may be interpreted as external electric fields, no current must exist.

## Conclusions

It has been shown that the primary structure of the protein molecule may be considered as a semiconductor nanowire in accordance with the numbers of filling representation. Based on this representation, it has been established that the conductivity band consists of five subzones. Three of these subzones have normal dispersion laws, while the rest two sub-bands have abnormal dispersion laws. Current density was calculated in the conditions of the complete absence of any disturbances of the electrostatic equilibrium of the system. It has been shown that under such conditions, current density is exactly equal to zero, and this fact is the evidence of correctness of the calculated conductivity band, as well as evidence of possibility to apply this calculated conductivity band in respect of the actual situation, which is connected with the presence of external fields.
